# Mediating effects of self-esteem on the relationship between mindful parenting and social anxiety level in Chinese adolescents

**DOI:** 10.1097/MD.0000000000032103

**Published:** 2022-12-09

**Authors:** Wu Chong-Wen, Li Sha-Sha, E Xu

**Affiliations:** a Department of Nursing, Huzhou Normal University, Zhejiang, China.

**Keywords:** adolescent, mindful parenting, self-esteem, social anxiety

## Abstract

This study aimed to investigate the relationship between mindful parenting and social anxiety level in Chinese adolescents and to explore the mediating roles of self-esteem between mindful parenting and social anxiety level. A total of 302 adolescents and their main caregivers were investigated by using the Chinese version of the Mindful Parenting Scale, Self-Esteem Scale and the Center for Epidemiological Studies Depression Scale and the Social Anxiety Scale. Related analysis was used to investigate the relationship between mindful parenting, self-esteem and social anxiety level. Mindful parenting and self-esteem were significantly associated with social anxiety level. Self-esteem mediated the relationship between mindful parenting and social anxiety level. Both mindful discipline and being in the moment influenced adolescents’ social anxiety level through self-esteem. Self-esteem completely mediated the association between mindful parenting and social anxiety level. Longitudinal research is needed to better understand the relationship between mindful parenting and social anxiety level in adolescents.

## 1. Introduction

According to the DSM-5, social anxiety disorder (SAD) refers to individuals who have an obvious fear or anxiety when faced with one or more social situations in which they are scrutinized by others. SAD is a relatively high prevalence of psychological problem among adolescents.^[1]^ In China, the proportion of adolescents with SAD was 23% to 46%.^[2]^ Adolescents’ psychological development lags behind their physical development, and the imbalance of their physical and mental development may affect their emotional, thinking and physical functions, and then they may face some psychological and social crisis, which makes it difficult to effectively deal with interpersonal communication, academic achievements and other problems.^[3]^ SAD in adolescents can significantly predict adverse psychological functioning later in life and seriously affect the healthy development of personality.^[4]^

Studies have shown that social anxiety disorder is a combination of physiological, psychological and social factors, and academic pressure, peer influence, family structure and other factors may be significant predictors of social anxiety disorder among adolescents.^[5]^ Compared with other influencing factors, parental rearing can better explain and predict adolescent psychological and behavioral problems.^[6,7]^ Some studies pointed out that parents’ rejection, excessive protection and lack of emotional warmth were related to the incidence of SAD in adolescents. On the other hand, paternal emotional warmth can reduce the incidence of SAD.^[8]^

“Mindful parenting” provides a new perspective and concept for parenting, which is a parenting style characterized by mindfulness.^[9]^ Mindful parenting refers to a non-judgmental parenting concept that is extremely open to the present self and the child. Parents bring such an accepting and well-meaning attitude into their relationship with their children.^[10]^ Researchers have divided mindful parenting into five dimensions: Listening with full attention to the child, emotional awareness of the self and the child, non-judgmental acceptance of the self and the child, self-regulation in the parenting relationship, and compassion for the self and the child.^[11]^ A recent study has shown that mindful parenting has a significant impact on parenting experience and emotional expression in parent-child conflict.^[12]^ The lower the level of mindful parenting, the weaker the emotional regulation ability of adolescents, and the more likely they are to produce negative emotions.^[13]^ Mindful parenting can effectively reduce parenting pressure and help parents to be aware of their children’s emotions, adjust their way of getting along with their children and effectively reduce the incidence of adolescent emotional disorders.^[12]^ At present, few studies investigate the relationship between mindful parenting and social anxiety level, especially in China. Therefore, this study proposed hypothesis H1: Parental mindful parenting level can negatively predict adolescent’ social anxiety level.

Studies have shown that low self-esteem was strongly correlated with SAD.^[14]^ Self-esteem is an individual’s overall evaluation and attitude towards their own value and status, and the degree to which they evaluate and accept themself. Studies have found that high self-esteem was associated with better positive performance, fewer emotional and behavioral problems.^[15]^ When individuals can correctly evaluate and accept themselves, they will feel a higher sense of value and self-esteem for their own existence, thus producing a more positive emotional experience. The formation and development of adolescent self-esteem are greatly influenced by childhood experience, which includes parental rearing style and peer relationship.^[16]^ Previous studies have revealed that authoritarian parenting style was significantly correlated with low self-esteem, while democratic or flexible parenting style was significantly correlated with high self-esteem.^[17]^ As a parenting style with acceptance, empathy and being in the present, mindful parenting was significantly positively correlated with self-esteem level of adolescents.^[18]^ Therefore, this study proposes hypothesis H2: self-esteem plays a mediating role between mindful parenting and adolescent’ social anxiety level.

According to the Mindful Parenting Scale (MIPQ) developed by McCaffrey et al,^[19]^ mindful parenting is divided into two dimensions: being in the moment and mindful discipline. Being in the moment represents the child-oriented aspects of mindful parenting, including present-focused attention, empathy and acceptance of the child. Mindful discipline represents the parent-oriented aspects of mindful parenting, including non-reactive parenting, nurturing awareness, and goal-oriented parenting. Being in the moment and mindful parenting represent two different aspects of mindful parenting. Therefore, it is necessary to explore whether these two different dimensions can predict social anxiety level and self-esteem of adolescents and play a mediating role in their respective prediction.

Geurtzen et al^[20]^ found that parents’ scores of “non-judgmental acceptance” in educating their children were significantly negatively correlated with their children’s anxiety level. Parent et al^[13]^ hold the view that when parents have non-evaluative and non-reactive awareness of their children, children are more able to express their feelings and thus reduce negative emotions. Moudgil and Moudgil^[21]^ found that when parents and children interact, the higher the present involvement, the more self-awareness children have. When parents compare their children, the frequency of comparison is significantly negatively correlated with adolescents’ self-esteem, that is, when parents tend to focus on others and make comparisons rather than the children themselves, the children will develop lower self-esteem. Therefore, the study proposed hypothesis H3: Being in the moment can negatively predict adolescent’ social anxiety level, and self-esteem plays a mediating role between being in the moment and social anxiety level.

Aremu et al^[17]^ confirmed that there was a positive correlation between flexible parenting style and self-esteem, and there was a negative correlation between autocratic and negligent parenting style and adolescents’ self-esteem. Gao et al^[22]^ pointed out that adolescents with authoritative or permissive parents had higher egos, and they felt respect and satisfaction with their lives. Peng et al^[23]^ found that both explicit and implicit self-esteem were negatively correlated with parents’ emotional warmth and understanding. Therefore, this study proposed hypothesis H4: Mindful discipline can negatively predict adolescent’ social anxiety level, and self-esteem plays a mediating role between mindful discipline and social anxiety level.

## 2. Materials and Methods

### 2.1. Study design

A cross-sectional study was conducted to investigate the relationship between mindful parenting of parents, self-esteem and social anxiety level of adolescents in China. The study was carried out in January to March 2022.

### 2.2. Participants and recruitment

The participants were recruited from two high schools in Zhejiang Province through an offline propaganda lecture which mainly introduces the aim of the study. Inclusion criteria were as follows: adolescents and their parents age under 18; under supervision by parents; and Both adolescents and parents are willing to participate. Adolescents with serious mental illnesses such as bipolar disorder and neurosis were excluded after diagnostic interview by a psychiatrist. Finally, 302 adolescents and their parents were recruited in the study.

### 2.3. Ethics and consent

This study was approved by the institutional review board at Huzhou Normal University and complies with the Helsinki Declaration. Written informed consent was obtained from all adolescents and their parents.

### 2.4. Measurement

Chinese version of the Mindfulness in Parenting Questionnaire (MIPQ): The 28-item Chinese version of MIPQ developed by Wu et al^[24]^ was used to measure parents’ mindful parenting level. It consists of 2 subcategories, including being in the present and mindful discipline. Each item is scored on a 4-point Likert scale ranging from 1 (not at all) to 4 (always). Higher scores indicated higher mindful parenting. This scale has been widely used among Chinese parents and has good reliability and validity.

Chinese version of The Rosenberg Self-Esteem Scale (SES): There were 10 items in the scale and a 4-point Likert scale ranging from 1 (very inconsistent) to 4 (very consistent) was adopted. The higher total score means the higher levels of self-esteem. This scale has been widely used among Chinese adolescents and has good reliability and validity.^[25]^

Interaction Anxiousness Scale: Interaction Anxiousness Scale was used to assess the tendency of subjective social anxiety experience of adolescents. There were 15 items in the scale and a 5-point Likert scale was adopted. The total score ranged from 15 (lowest social anxiety) to 75 (highest social anxiety). This scale has been widely used among Chinese adolescents and has good reliability and validity.^[26]^

### 2.5. Data collection

An online survey platform (https://www.wjx.cn) in China was used in this study. The online survey link was sent by the researcher to all adolescents and their parents who were willing to participate the study. All data were collected by the WenJuanXing program. In order to improve the accuracy of questionnaire response, the electronic questionnaire was equipped with logic check, error reminder, automatic jump and other verification procedures.

### 2.6. Statistical analysis

The Statistical Packages for Social Sciences, version 25 (IBM Corp., Chicago, IL) was used to analyze the data. The mean and standard deviation of mindful parenting, self-esteem and social anxiety levels were analyzed by descriptive statistics. Pearson’s correlation test was used to investigate the relationship among mindful parenting, self-esteem and social anxiety levels. The mediation effect test model of Bootstrap was used to test and analyze the mediating effect of self-esteem whether played a mediating role between mindful parenting and social anxiety levels. A *P* value ≤ .05 was considered statistically significant.

## 3. Results

### 3.1. Description of mindful parenting, mindful discipline, being in the present, self-esteem, and social anxiety

The average scores of mindful parenting, mindful discipline, being in the present, self-esteem, and social anxiety were found to be 73.69 ± 17.46, 34.81 ± 8.87, 38.88 ± 9.80, 28.69 ± 3.40, and 40.91 ± 8.74, respectively.

### 3.2. The relationship between mindful parenting, mindful discipline, being in the present, self-esteem, and social anxiety

There was a significant negative correlation between self-esteem and social anxiety level of adolescents, and they were significantly correlated with the two sub-dimensions of parents’ level of mindful parenting. Details are shown in Table 1.

**Table 1:** Analysis of the correlation between mindful parenting (including two dimensions), self-esteem and social anxiety (n = 303).

Variables	Being in the present	Mindful discipline	Mindful parenting	Self-esteem	Social anxiety
Being in the present	1				
Mindful discipline	0.748*	1			
Mindful parenting	0.928*	0.942*	1		
Self-esteem	0.304*	0.323*	0.336*	1	
Social anxiety	−0.407*	−0.425*	−0.445*	−0.415*	1

* *P* < .001.

### 3.3. Mediating effect of self-esteem

#### 3.3.1. The mediating effect of self-esteem on mindful parenting and social anxiety.

In the regression analysis, we set social anxiety as the dependent variable and mindful parenting as the independent variable and self-esteem as the mediating variable. The results are shown in Table 2. The results showed that self-esteem mediated the effects of mindful parenting on social anxiety levels, with an explanatory power of 22.73%.

**Table 2:** Mediating analyze of self-esteem on mindful parenting on social anxiety (N = 303).

	β	SE	95% CI		Account
			Lower	Upper	
Total effect	−0.223	0.025	−0.274	−0.172	
Direct effect	−0.173	0.026	−0.224	−0.121	77.27%
Indirect effect	−0.050	0.013	−0.077	−0.027	22.73%

#### 3.3.2. The mediating effect of self-esteem on mindful discipline and social anxiety.

In the regression analysis, we set social anxiety as the dependent variable and mindful discipline as the independent variable and self-esteem as the mediating variable. The results are shown in Table 3. The results showed that self-esteem mediated the effects of mindful discipline on social anxiety levels, with an explanatory power of 23.75%.

**Table 3:** Mediating analyze of self-esteem on mindful discipline on social anxiety (N = 303).

	β	SE	95% CI		Account
			Lower	Upper	
Total effect	−0.379	0.047	−0.471	−0.287	
Direct effect	−0.289	0.046	−0.382	−0.198	76.25%
Indirect effect	−0.090	0.023	−0.138	−0.046	23.75%

#### 3.3.3. The mediating effect of self-esteem on being in the present and social anxiety.

In the regression analysis, we set social anxiety as the dependent variable and being in the present as the independent variable and self-esteem as the mediating variable. The results are shown in Table 4. The results showed that self-esteem mediated the effects of being in the present on social anxiety levels, with an explanatory power of 23.94%.

**Table 4:** Mediating analyze of self-esteem on being in the present on social anxiety (N = 303).

	β	SE	95% CI		Account
			Lower	Upper	
Total effect	−0.401	0.052	−0.503	−0.299	
Direct effect	−0.305	0.051	−0.406	−0.203	76.06%
Indirect effect	−0.096	0.026	−0.150	−0.048	23.94%

## 4. Discussion

The results of this study showed that parents’ level of mindful parenting, mindful discipline and being in the present have the negative relationship with adolescents’ social anxiety level and have the positive relationship with adolescents’ self-esteem. The mindful parenting of parents plays an important role in adolescents’ internalization problems. Parent et al^[13]^ conducted a survey on adolescents at different ages and found that the level of mindful parenting of parents was negatively correlated with their negative parenting style, while the negative parenting style was significantly positively correlated with adolescents’ internalization problems. In other words, the lower level of parents’ mindful parenting will produce higher negative parenting behaviors, which will make adolescents more prone to depression, anxiety and other psychological problems. Bogels and Restifo^[27]^ found that parents who learned mindfulness techniques experienced less parenting stress and impulsivity, as well as significantly improved co-parenting and marital relationships, which were strongly correlated with parenting quality and adolescent psychological problems. Geurtzen et al^[20]^ proved that the six dimensions of mindful parenting and the overall quality of mindful parenting were associated with internalizing problems such as anxiety in adolescents, when controlling for traditional parenting dimensions.

The results showed that there was a significant positive correlation between the two dimensions of parental mindful parenting and the level of self-esteem. In other words, the higher the level of mindful parenting, the higher the self-esteem of adolescents. Aremujohn-akinola and Desmennu^[17]^ found a high correlation between parenting style and self-esteem. At the same time, the flexible democratic parenting style was significantly correlated with self-esteem, while authoritarian, evasive and indulgent parenting style were negatively correlated with self-esteem level of children. The non-evaluative and present-focused characteristics of mindful parenting are obviously consistent with the concept of democratic parenting. Being in the present dimension represents the child-oriented parenting point, including focusing attention on the present moment, acceptance and empathy for children in all aspects; Mindful discipline represents parent-oriented aspects, including non-responsiveness to child behavior, parenting awareness, and goal-oriented parenting.^[19]^ This attention and acceptance will make children have a more positive and comprehensive understanding of themselves and build confidence in daily life, so that children can have a higher level of self-esteem.

The results showed that there was a significant negative correlation between self-esteem and social anxiety. Empirical studies have also found that people with low self-esteem pay too much attention to negative evaluation from others and are more sensitive to rejection from others, which leads to the increase of anxiety level in social situations.^[28]^ Individuals with higher self-esteem have a clearer self-concept and can form correct attribution and explanation for negative evaluation from others in social activities and are less prone to anxiety.^[29]^ People with low self-esteem have negative self-evaluation. Such negative beliefs are stored in memory and automatically activated in social situations, resulting in an incorrect understanding of the situation and strong social anxiety.^[30]^

Moreover, the current study showed that the level of mindful parenting of parents can negatively predict adolescent’ social anxiety level through self-esteem. Pelham and Swann^[31]^ divided overall self-esteem into three main influencing factors: the tendency to experience positive and negative emotional states, specific self-views, and the way in which they construct their self-views. Adolescents’ self-esteem was significantly influenced by these three factors. It can be seen from the results of the study that the level of mindful parenting of parents can significantly predict the level of adolescent anxiety in general, which was consistent with the results of other studies.^[12,32]^ Mindful parenting refers to parents’ non-responsiveness and parenting awareness towards their children. Focusing on the present represents parents’ actual acceptance, attention and corresponding communication with their children. The direct effect of mindfulness on children’s mental health may be smaller than that of present-focus, which may explain why mindfulness was less of a direct predictor of adolescent anxiety than present-focus in the analysis. At the same time, as a kind of education pattern, mindful parenting not only directly affects the parents in the children’s education. In practical parenting, parents’ attention, acceptance and empathy to their children help children to correctly understand themselves, to establish a harmonious family environment. This process will affect children’s self-esteem, and then relieve individual emotions, thus reducing anxiety and other negative emotions.

## 5. Strengths and limitations

To our best knowledge, this is the first study to explore the mindful parenting-self-esteem-social anxiety link among Chinese adolescents. However, there were still limitations to this study. Firstly, the participants of this study were recruited from two high schools, so readers need to raise caution when applying our findings to adolescents from other countries or from different cultural backgrounds. Further studies should be conducted on larger samples in different cultures. Moreover, the self-report nature of the measures means that responses were subject to bias and socially desirable responding.

## 6. Conclusions

This study confirmed the mediating effect of self-esteem between mindful parenting and social anxiety in adolescents. The results of this study could help researchers better understand the relationship between mindful parenting of parents and self-esteem and social anxiety levels of adolescents. It is suggested that mindful parenting could be a means to improve the self-esteem of adolescents and thus prevent SAD in adolescents.

## Acknowledgments

All authors were grateful for all the participants and all student counselors in this study for their cooperation.

## Author contributions

**Conceptualization:** Wu Chong-Wen, E Xu.

**Data curation:** Wu Chong-Wen, E Xu.

**Formal analysis:** Wu Chong-Wen, E Xu, Li Sha-Sha.

**Investigation:** Wu Chong-Wen, E Xu, Li Sha-Sha.

**Methodology:** Wu Chong-Wen, E Xu, Li Sha-Sha.

**Software:** Li Sha-Sha.

**Writing – original draft:** Wu Chong-Wen, E Xu, Li Sha-Sha.

**Writing – review & editing:** Wu Chong-Wen, E Xu, Li Sha-Sha.
